# A Multicentre Analysis of Approaches to Learning and Student Experiences of Learning Anatomy Online

**DOI:** 10.1007/s40670-022-01633-7

**Published:** 2022-09-19

**Authors:** Danya Stone, Georga J. Longhurst, Kate Dulohery, Thomas Campbell, Annalise Richards, Dominic O’Brien, Thomas Franchi, Samuel Hall, Scott Border

**Affiliations:** 1 Department of Medical Education, Brighton and Sussex Medical School, Brighton, East Sussex, BN1 9PX UK; 2 Department of Anatomical Sciences, St George’s University of London, London, SW17 0RE UK; 3 School of Medicine, University of Sunderland, Sunderland, SR1 3SD UK; 4 School of Medicine, University College Dublin, Dublin, D04 V1W8 Ireland; 5 School of Anatomy, University of Bristol, Bristol, BS8 1QU UK; 6 School of Medicine, University of Sheffield, Sheffield, S10 2TN UK; 7 Centre for Learning Anatomical Sciences, Southampton University, Southampton, SO17 1BJ UK; 8 Department of Anatomy, University of Glasgow, Glasgow, G12 8QQ UK

**Keywords:** Anatomy education, Approach to learning, COVID-19, Remote learning, Distance learning, Blended learning

## Abstract

**Introduction:**

The approaches to learning students adopt when learning anatomy online could yield important lessons for educators. Dissection room teaching can encourage students to adopt a deep approach to learning anatomy. It was therefore hypothesized that the proportion of students adopting a deep approach to learning would be lower in a population learning anatomy online. This research aims to investigate the experiences of students learning anatomy online during the COVID-19 pandemic and the approaches to learning they adopted.

**Methods:**

A survey was distributed to medical students at 7 universities across the UK and Ireland. The survey included two previously validated questionnaires: Approaches and Study Skills Inventory for Students and Anatomy Learning Experience Questionnaire.

**Results:**

The analysis included 224 unique student responses. Students’ approach to learning mirrored reports from previous studies conducted during face-to-face tuition with 44.3% adopting deep, 40.7% strategic, 11.4% surface, and 3.6% combined learning approaches. The university (*p* = 0.019) and changes to formative (*p* = 0.016) and summative (*p* = 0.009) assessments significantly impacted approach to learning. Students reported that online resources were effective but highlighted the need for clearer guidance on how to find and use them successfully.

**Conclusion:**

It is important to highlight that students value in-person opportunities to learn from human cadaveric material and hence dissection room sessions should remain at the forefront of anatomical education. It is recommended that future online and/or blended provisions of anatomy teaching include varied resources that maximize engagement with media featuring cadaveric specimens.

**Supplementary Information:**

The online version contains supplementary material available at 10.1007/s40670-022-01633-7.

## Introduction

The COVID-19 pandemic resulted in the suspension of in-person teaching globally, affecting an estimated 91% of the student population [1]. Advances in modern technology and the widespread availability of the Internet allowed education to continue, albeit, in an online format. The pandemic can provide important insight into students’ experiences of online learning and the challenges associated with this. With student numbers continuing to rise (the Medical Schools Council recommend the number of medical students in the UK should increase by 5000 [2]) and constant advances in technology, it is likely that universities will continue to incorporate online delivery into their teaching and assessment provisions. Online education during the COVID-19 pandemic can, therefore, provide a valuable opportunity to reflect on the balance of in-person and online teaching and assessment practices offered by universities going forward.

In anatomy, the delivery of teaching and assessments through online formats is associated with a specific set of challenges [3]. For example, anatomy syllabi traditionally feature practical classes, during which students learn utilizing multiple resources simultaneously, making them difficult to reproduce in an online format. Moreover, these resources often include the use of human cadaveric specimens [3–7] that should have explicit consent and secure platforms in place if images or videos of these specimens are shared online [8]. In addition, by the very nature of this three-dimensional subject, it is difficult to portray important anatomical relations and variations through a computer screen [3, 9]. It is therefore of interest for anatomical educators to determine whether an online anatomy provision can be effective and whether it can still inspire the adoption of deep approaches to learning the subject.

### Online Anatomy Teaching During the COVID-19 Pandemic

The resources and methods utilized to teach anatomy online during the COVID-19 pandemic have been previously described by a myriad of reports [2, 10–25]. The application of these resources has been influenced by the digital learning infrastructure available to educators [2, 16, 26]. Alongside the online delivery of synchronous and asynchronous lectures, many anatomists created in-house digital resources using cadaveric specimens and models. Moreover, anatomists utilized commercially available tools such as three-dimensional (3D) virtual models and other existing digital content, including massive online open courses (MOOCs) and anatomy YouTube videos [2, 11, 19] and even virtual reality programs [19, 27].

### Online Anatomy Assessments During the COVID-19 Pandemic

There are several accounts of changes made to assessment in anatomical education during the COVID-19 pandemic in Asia [11, 28, 29], America [18, 30], Europe [9, 31] and Oceanica [16]. Some universities adopted an open book approach whereby “flag randomization” was used for spotter questions, with questions testing higher-order thinking incorporated to reduce the potential for student collusion [16]. Other universities opted to postpone or cancel planned examinations or convert them to a formative process [10]. Assessments allow students to monitor their own learning and act as important motivators that ultimately improve understanding, especially if a feed-forward approach is implemented by educators [32–34]. Hence, it is reasonable to hypothesize that changes to assessments made during the pandemic will impact on learning.

### Altered Approaches to Learning During the COVID-19 Pandemic

The Approaches and Study Skills Inventory for Students (ASSIST) questionnaire has been used to explore three main constructs of learning approaches; deep, strategic and surface [35–37]. Students adopting a deep approach to learning tend to be intrinsically motivated to seek meaning from the topic, draw their own conclusions and are able to monitor the effectiveness of their own learning. In contrast, students adopting a strategic approach to learning tend to be organized with good time management skills and are normally motivated to achieve high scores in assessments. Students adopting a surface approach to learning often find little immediate purpose or interest in their learning. They utilize rote learning techniques and may not understand concepts in-depth. Therefore, they rarely excel beyond meeting the minimum requirements to pass their examinations. Students adopting a surface approach to learning are usually extrinsically motivated by fear of failure [37, 38].

Learning approaches are not fixed and can be dependent upon the context in which learning takes place. Course design has been shown to influence students’ approach to learning. This is multifaceted and includes the taxonomy of learning objectives [39], the nature of feedback and the assessment design [40]. During the COVID-19 pandemic, fundamental changes were made to course design, and therefore, an analysis of approach to learning can be used to provide an insight into the overall impact on student engagement in their anatomy learning online.

Smith and Mathias [41] adapted the ASSIST to identify how students approach learning anatomy. This has been used in conjunction with another bespoke survey; the anatomy learning experiences questionnaire (ALEQ) to establish that the context of anatomical learning, such as local curriculum factors, can impact the approach students adopt to learn anatomy [5, 42].

Factors that encourage a deep approach to learning include the student’s ability to use their hands to palpate anatomical structures in the dissection room, to verbalize anatomical terminology and to apply their knowledge to clinical scenarios [5]. It is, therefore, reasonable to hypothesize that with the suspension of dissection room teaching, and with a lack of opportunities for medical students to consolidate their anatomical knowledge in clinical settings, there will be a limited number of students adopting a deep approach to learning. Additionally, large-scale changes in assessment methodology lead us to hypothesize there will be a reduction in students’ motivation to learn anatomy and this will limit the number of students adopting a strategic approach to learning. Thus, understanding approaches to learning during the pandemic could yield important lessons for educators when making decisions surrounding blended delivery of curricula.

### Aims

The aims of this study were threefold:

- To evaluate the experience of medical students while learning anatomy online
- To explore medical students’ perspectives of the effectiveness of educational tools utilized to teach anatomy online at their institution
- To investigate the approach to learning students adopted while learning online during the COVID-19 pandemic

## Materials and Methods

### Ethics

Ethical approval was granted by Brighton and Sussex Medical School (BSMS) Research Governance Ethics Committee (ER/BSMS9GHM/1), and thereafter gatekeeper approval was provided by the other six participating universities.

### Survey Instrument

An online survey was designed using Qualtrics survey software. The survey consisted of two previously validated Likert scale questionnaires: the ASSIST [37] and ALEQ [5]. The ALEQ was adapted to specifically capture the perceptions of learning anatomy online, informed by previous research by Longhurst et al. [2]. This resulted in a 33-item adapted ALEQ. An additional 12 multiple-choice questions and three open-ended questions were designed to add context to the shift in learning environments experienced by the students. This resulted in a 100-item survey that was pilot tested by a sample group of colleagues and medical students (*n* = 9).

### Context

Students from a convenience sample of seven universities across the UK and Ireland were surveyed: Brighton and Sussex Medical School, St George’s University of London, University of Birmingham, the University of Bristol, University College Dublin, the University of Southampton and the University of Sunderland. Prior to data analysis, each university was assigned a number (one to seven) to maintain inter-institutional anonymity of results. Before the COVID-19 pandemic, all but one of these universities delivered anatomy syllabi via face-to-face practical sessions using either cadaveric dissection, prosections, or a combination of both ( Table 1). The final university never taught using cadaveric specimens. Of the universities that taught using cadaveric material, the number of hours dedicated to dissection room teaching ranged from 4 h a term to 4 h a week. During the COVID-19 pandemic, four universities delivered summative assessments virtually, while the rest cancelled the summative assessment for that year. Formative anatomy assessments were delivered by five of the universities prior to the COVID-19 pandemic, of these, four delivered their formative assessments online, while one institution decided to cancel their formative assessment (Table 1).

**Table 1:** Universities teaching and assessment in anatomy before and during June/July 2020. Authors at each institution provided information on how cadaveric specimens were used to teach anatomy prior to the COVID-19 lockdown and how much exposure students normally received to cadaveric material (either per week or per term depending

University	Method of teaching using cadaveric specimens (dissection/prosection)	Normal exposure to cadaveric material	Formative assessment cancelled?	Summative assessment cancelled?
1	Both	3 h/week	No	No
2	Both	1.5–3 h/week	No	Yes
3	Prosection	4 h/term	No	Yes
4	Prosection	1.5 h/week	Yes	No
5	None	0	-	No
6	Prosection	8–10 h/term	No	Yes
7	Both	4 h/week	No	No

### Inclusion criteria

Participants were included in this study if they attended one of the seven forementioned universities as a medical student and were completing an anatomy module taught online during June and July of 2020. All participants who met the inclusion criteria at the author’s institutions were contacted by email and invited to complete the survey. Medical students in years one and two at all of the universities met the inclusion criteria, and hence all students from these year groups were included. At universities where anatomy teaching is also delivered in other years (year 3 or during an intercalated degree), these students also received the invite to participate. In total, the invitation to complete the survey was sent out to 3,516 students.

### Analysis

Frequency distributions and statistical analyses of the data were performed using the Statistical Package for Social Sciences (SPSS) for Windows, version 22 (IBM Corp., Armonk, NY).

As multiple analyses were performed, the specific statistical test utilized is stated alongside the result. Values of *p* ≤ 0.05 were considered statistically significant. All statistically significant results are included in Tables 2 to 3. The approach to learning for each participant was calculated as detailed in the revised ASSIST inventory [37]. Kruskal–Wallis tests were used to determine the relationship between the predominant approach to learning for each student and responses to the Likert questions of the ALEQ.

Free-text responses underwent reflexive thematic analysis according to the method described by Braun et al. [43]. An inductive, semantic and realist approach was adopted. All responses were read in order to ensure familiarity with the dataset. The responses were subsequently analysed and assigned codes relating to the topics covered. The assigned codes were collated, and common themes were observed and agreed upon by all authors.

## Results

### Demographics

Of 3,516 students, from the seven universities, who met the inclusion criteria, 242 unique student responses were received (6.9% response rate). Data from eighteen respondents were excluded as they did not meet the specified inclusion criteria, resulting in a final dataset of 224 respondents. Incomplete responses remained in the analysis, and therefore, the total number of responses varies between questions.

Of the 224 medical students included in the final analysis, 180 (80.5%) had no prior degree, and 44 (19.6%) had completed a degree prior to studying medicine (Table 2). One hundred and thirty-six (60.7%) were first year students, 77 (34.4%) were second year students, nine (4.0%) were third year students, and two (0.9%) were intercalating.

**Table 2:** Demographics of students surveyed. All medical students learning anatomy during the survey period were included in the inclusion criteria; hence, the years of study sampled vary between institutions

		**Year**				**Previous study**	
University	**Number of responses (response rate %)**	**1**	**2**	**3**	**Intercalating**	**No prior degree**	**Prior degree**
1	60 (7.5)	43	17	0	0	60	0
2	10 (2.0)	1	9	0	0	8	2
3	28 (5.6)	12	14	0	2	27	1
4	34 (7.1)	27	7	0	0	21	13
5	13 (26.0)	13	0	0	0	13	0
6	31 (5.4)	20	11	0	0	19	12
7	48 (7.8)	20	19	9	0	32	16
Total	**224 (6.4)**	**136**	**77**	**9**	**2**	**180**	**44**

### The Impact of Online Learning on Approaches to Learning

**Fig. 1:**
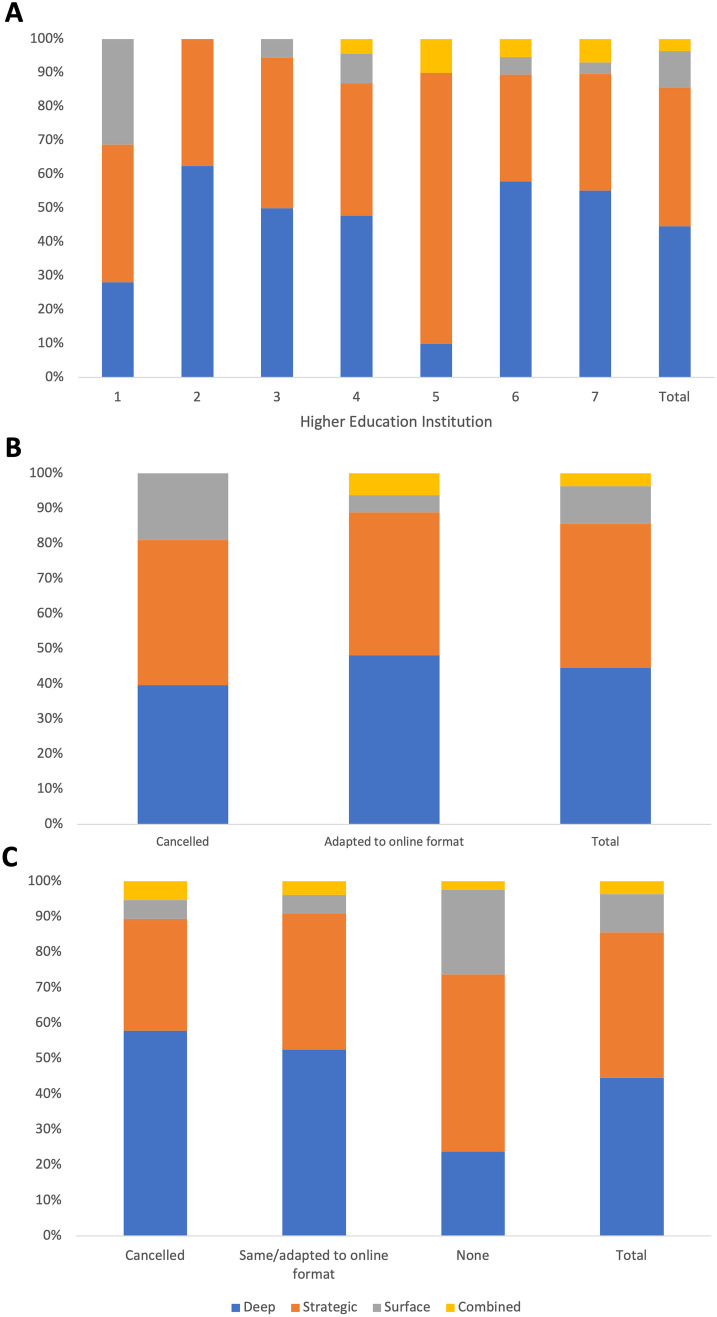
Relationship between approach to learning and curriculum factors. **A** The proportion of students that adopted each approach to learning at universities 1–7. **B** The proportion of students adopting each approach to learning where summative assessments were canceled or adapted to an online format. **C** The proportion of students adopting each approach to learning dependent on changes made to formative assessments. The total column represents the overall proportion of learners adopting each approach to learning

One hundred and forty responses were included in the ASSIST analysis. The majority of students adopted a deep approach to learning anatomy online (*n* = 62, 44.3%). Fifty-seven students (40.7%) adopted a strategic approach, while 16 (11.4%) adopted a surface approach. Only five students (3.6%) adopted a combined approach (Fig. 1A). A chi-squared test for independence revealed that the university attended, in addition to changes to summative (Fig. 1B) and formative assessment (Fig. 1C), were statistically significant factors affecting approach to learning [*X*^2^ (18, *n* = 139) = 32.6, *p* = 0.019; *X*^2^ (3, *n* = 139) = 10.3, *p* = 0.016; *X*^2^ (6, *n* = 139) = 17.1, *p* = 0.009 respectively]. All other characteristics (year of study, age, previous study, additional hours spent studying) did not significantly affect the approach to learning anatomy online (Table 3).

**Table 3:** Relationship between approach to learning and Likert scale questions from the Anatomy Learning Experience Questionnaire (ALEQ). A Kruskal–Wallis test was used to determine any significant relationships between the approaches to learning adopted and agreement with all Likert scale questions included in the Anatomy Learning Experiences Questionnaire (ALEQ). Results showing a significance level of *p* ≤ 0.05 are reported here. All non-significant results can be found in Table 6

Question	Significance (P valve)	Modal response			
		**Deep**	**Strategic**	**Surface**	**Combined**
I find/found non-cadaveric videos sourced online (e.g. YouTube) an effective way of learning anatomy	0.008	Somewhat agree	Agree	Agree/ somewhat agree	Somewhat disagree
I believe/d that the anatomy resources offered online by the school are limited	0.013	Agree	Somewhat agree	Disagree	Somewhat agree
The most effective way I learnt anatomy in the dissecting room was to get my hands in and feel for structures	0.027	Agree	Agree	Somewhat agree	Agree

### Experience of Using Online Anatomy Educational Resources

**Fig. 2:**
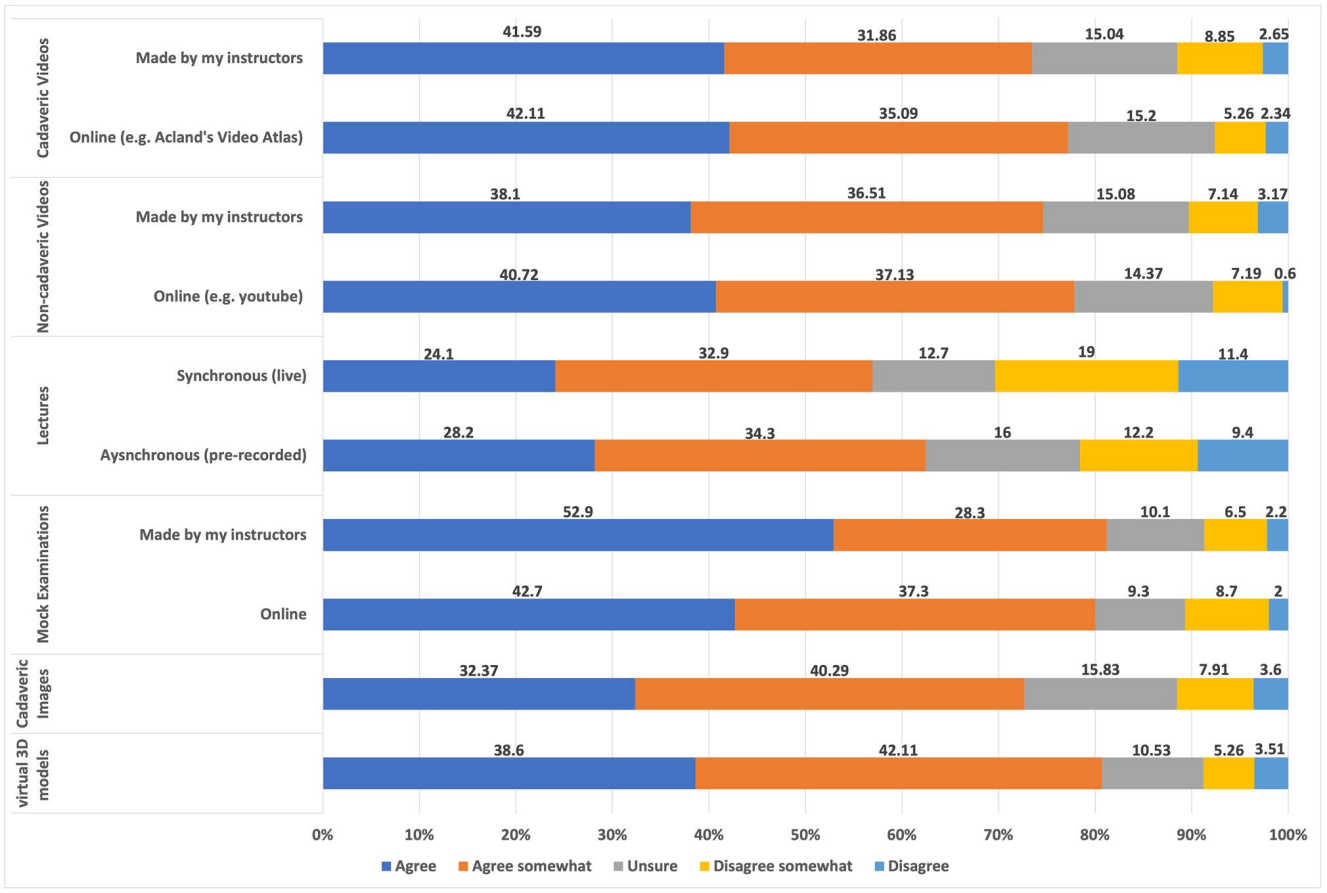
Students’ perceptions of online learning resources. Students responded to the Likert statement: “I find/found [learning resource] to be an effective way of learning anatomy”. *The perceived effectiveness was significantly impacted by the university students attended. + Significant difference between resources made by instructor and resources sourced online

Kruskal–Wallis tests revealed the university students attended statistically impacted the perceived effectiveness of the following resources; online cadaveric video (*p* = 0.049), instructor made cadaveric videos (*p* = 0.035), virtual 3D models (*p* = 0.048) and instructor made mock examinations (*p* = 0.05) (Fig. 2) (for full details, see Table 4). Opinions of all other resources did not vary significantly between students attending each university (cadaveric images, non-cadaveric videos either found online or provided by instructor, synchronous and asynchronous lectures and online mock examinations).

**Table 4:** Dependence of approach to learning. A chi-square test of independence was performed to examine the relationship between the context of learning, determined through answers to Likert questions shown above, and the approach to learning students are adopting. *Significant results (*p* ≤ .05) are reported in text

Question	Significance (*p* value)
At which higher education institution are you studying?	0.019*
Have you completed a degree prior to studying medicine?	0.330
What year of your studies are you currently undertaking?	0.812
What was the format of your summative exam during COVID-19?	0.016*
What was the format of your formative exam during COVID-19?	0.009*
Approximately how many hours of additional study did you spend studying anatomy during COVID-19?	0.906

A chi-squared test demonstrated that students had a clear preference for mock examinations created by their instructors compared to assessments sourced online [*X*^2^ (4, *n* = 113) = 21.74, *p* = 0.0002]. Further chi-square tests revealed no significant differences between student preferences for asynchronous or synchronous lectures and videos (cadaveric and non-cadaveric) made by the instructor or sourced online (Fig. 2).

### Approach to Learning and Responses to the ALEQ

Kruskal–Wallis tests were used to determine the relationship between the predominant approach to learning for each student and responses to the Likert questions of the ALEQ. Only results in which there was a statistical difference between approach to learning and the answers to the Likert questions are reported (Table 5) (for non-significant results, see Table 6). Kruskal–Wallis tests revealed that a significantly larger proportion of students adopting either a strategic or surface approach agreed or somewhat agreed that “non-cadaveric videos found online are effective methods to learn anatomy” [*X*^2^ (3, *n* = 114) = 8.189, *p* = 0.042] (Table 5). Surface learners were significantly less likely to agree that getting one’s “hands in and feeling for structures” is an effective way to learn anatomy [*X*^2^ (3, *N* = 121) = 9.202, *p* = 0.027]. Students who adopted a deep approach were significantly more likely to agree that “the anatomy resources offered online by the school are limited” [*X*^2^ (3, *n* = 131) = 10.699, *p* = 0.01] (Table 5).

**Table 5:** Relationship between the university students attended and responses to Likert scale questions. Kruskal–Wallis tests were performed to determine significant relationships between Likert questions and university. For each significant result (reported here) the modal answer to the Likert question is reported

		Modal Response						
**Question**	***p* value**	1	2	3	4	5	6	7
I find/found cadaveric videos found online (e.g. Acland’s Video Atlas) an effective way of learning anatomy	0.049	Agree	Somewhat agree	Agree	Agree	Agree	Agree	Somewhat agree
I find/found cadaveric videos made by my instructors an effective way of learning anatomy	0.035	Unsure	Agree	Agree	Agree	Somewhat agree	Somewhat agree	Agree
I find/found virtual 3D models an effective way of learning anatomy	0.048	Agree	Somewhat agree	Somewhat agree	Somewhat agree	Somewhat agree	Somewhat agree	Agree
I find/found online mock examinations made by my instructor an effective way of learning anatomy	0.050	Somewhat agree	Somewhat agree	Agree	Agree	Agree	Agree	Somewhat agree
I believe/d that the anatomy resources offered online by the school are limited	0.000	Agree	Agree	Somewhat agree	Somewhat agree	Disagree	Agree	Agree

**Table 6:** Relationship between the approach to learning students adopt and responses Likert scale questions. Kruskal–Wallis results showing a significant level between approach to learning and Likert scale rating to questions from the Anatomy Learning Experiences Questionnaire.*Significant results (*p* ≤ .05) are reported in the text

**Question**	Significance (*p* value)
I find/found dissection notes that have been adapted to a virtual format e.g. to include virtual dissection using 3D software an effective way of learning anatomy	0.142
I find/found dissection notes that have not been adapted to a virtual format e.g. notes available online that have not been adapted, an effective way of learning anatomy	0.937
I find/found cadaveric videos found online (e.g. Acland’s Video Atlas) an effective way of learning anatomy	0.089
I find/found cadaveric videos made by my instructors an effective way of learning anatomy	0.265
I find/found additional cadaveric images made/distributed by my instructors an effective way of learning anatomy	0.529
I find/found non-cadaveric videos found online (e.g. YouTube) an effective way of learning anatomy	0.042*
I find/found non-cadaveric videos made by my instructor an effective way of learning anatomy	0.240
I find/found virtual 3D models an effective way of learning anatomy	0.508
I find/found online mock examinations found online an effective way of learning anatomy	0.466
I find/found online mock examinations make by my instructor an effective way of learning anatomy	0.347
I find/found prerecorded lectures an effective way of learning anatomy	0.072
I find/found live lectures an effective way of learning anatomy	0.315
The most effective way I learnt anatomy in the dissecting room was to get my hands in and feel for structures	0.027*
The most effective way I learnt anatomy in the dissecting room was in groups	0.825
I felt the dissecting room was a daunting environment to learn in	0.568
I feel that I learned other things while in the dissecting room (e.g. natural variation)	0.186
I feel the lack of dissection room sessions will negatively affect my anatomy learning	0.354
Dissection room sessions were a good opportunity to ask questions	0.145
I find/found the amount of anatomy I need/ed to learn daunting	0.759
I believe/d that the anatomy resources offered online by the school are limited	0.013*
I have/had problems learning anatomy because I don't see the point to it	0.202
I have/had problems learning anatomy because the teaching styles do not suit me	0.120
I feel/felt the current course assessments do not reflect the learning that occurs	0.312
My main motivation for learning anatomy is/was (normally) to pass exams	0.931
I find/found anatomy learning difficult because it is memorization based	0.423
I feel/felt motivated to learn anatomy	0.746
I find/found I have less opportunity to use anatomical terms and language during lockdown	0.619
I find/found I have less opportunity use my anatomy radiology knowledge during lockdown	0.437
I find/found I have less opportunity to use my surface anatomy knowledge during lockdown	0.220
I find/found that my anatomy learning informs other subject learning	0.620
I feel/felt that understanding anatomy is a very important part of becoming a doctor	0.684
I feel/felt that working with cadaveric material is an important part of becoming a doctor	0.491
My opinions of anatomy's relevance have decreased during the COVID-19 lockdown	0.297

### Recommendations from Students for Online Anatomical Education

Responses (*n* = 163) to the following open-ended questions were thematically analysed: “Thinking about the resources offered by your anatomy instructors. Are you happy with the resources offered? Are there ways these could be improved?”.

Initially, seven themes were identified from these responses, which were further analysed and distilled into three main themes for more meaningful comparison. The three main themes identified were as follows: (1) no ‘one-size-fits-all' and hence a range of resources are required; (2) clear guidance improves the effectiveness of online resources; and (3) students desire maximum exposure to cadaveric material.

#### No ‘One-Size-Fits-All'

There was disagreement among students about the effectiveness of online anatomy resources offered by their institution. This disparity is echoed in comments around the effectiveness of specific resources. For example, in reference to the use of videos, one student stated:

*“I enjoyed the online videos [of cadaveric demonstrations] provided to us instead of cadaver labs. It was helpful that we were able to pause the videos to take notes”.*

While another said:

*“I think [my institution] has done well offering learning resources. But no substitute for the real thing. Practical sessions is how one learns anatomy. You can’t be a doctor by watching lectures and videos online”.*

In reference to the use of 3D software, one student said:

*“Using 3D anatomy software doesn't add anything to the anatomy lectures that we receive because it's just like looking at a diagram”.*

While another said:

*“I'm extremely satisfied with the 3D anatomy model software because it really helped me visualize everything”.*

#### Clear Guidance Improves Effectiveness of Online Resources

Participants stated that guidance was necessary to improve the effectiveness of online resources. This could include signposting, better integration of resources into the curriculum, ensuring resources are organized within the Virtual Learning Environment (VLE) and advice about the expected level of knowledge, particularly as online resources are not always tailored to the curriculum:

*“Resources are often tucked away on module pages that may be hard to find. The organisation of the anatomy web page makes looking for resources difficult and often frustrating. The resources that are there including complete anatomy, clinical key and Acland’s anatomy are good, but are rarely integrated to the curriculum”.*

*“[...] it was easy to miss and not notice useful resources. The resources could sometimes be clearer what is extra knowledge and what is essential, as clinical components and contextualization really helps for learning”.*

#### Students Desire Maximum Exposure to Cadaveric Material

Students viewed the loss of exposure to cadaveric material as detrimental, and there was a clear concern among students about the potential negative impact that loss of time in the dissection room may have on their studies and confidence in anatomy.

One comment summarises the general feeling among respondents:

*“We were deprived of the opportunity to dissect which based on my prior experience of learning anatomy is the cornerstone in consolidating anatomy learning. I believe the only way it could be improved is to return students to the dissection lab”.*

Although many comments referred specifically to the laboratory environment, many students also discussed the desire for more online exposure to cadaveric material, particularly videos of dissection, or images of specimens:

*“[…] there needs to be a better replacement for our cadaver labs such as cadaveric videos that allows us to better observe”*

In general, respondents were adamant that nothing could replace the experience of practical classes. The students felt their dissection skills, and confidence identifying structures was impacted:

*“Pictures of specimens cannot and will never be able to replace the lab experience in my mind and so any student who undertakes ONLY online anatomy will struggle compared to one who studies cadaveric anatomy”.*

## Discussion

Students faced a seismic shift in the delivery of their teaching during the COVID-19 pandemic, transitioning from traditional face-to-face learning to remote, online delivery of the material [26]. While this was a turbulent period for the education sector, it provides a unique opportunity to investigate the impact of online learning which is likely to continue in the future. The results discussed here represent the largest multi-centred survey of approaches to learning across seven universities. Smith and Mathias [41] demonstrated that under normal learning conditions, strategic and deep learners achieve higher scores compared to surface learners, which is also substantiated elsewhere in the literature [41, 44–46]. Therefore, understanding how students’ approach to learning was affected during the COVID-19 pandemic is a vital component in this decision-making process moving forward.

### Approaches to Learning Anatomy Online

The proportion of students adopting a deep approach to learning reported here (44.3%) is similar to results from previous studies that analysed students’ approach to learning anatomy in traditional face-to-face formats [5, 41, 42, 45, 47, 48]. Our results suggest that learning anatomy online does not limit the number of students adopting deep approaches to learning and might suggest that students remained internally motivated and interested in anatomy education, despite the shift in delivery to an online format. This should reassure academics that the incorporation of blended curricula will not immediately influence students to adopt or convert to a surface approach to learning, particularly if students are given appropriate resources and guidance [2, 46, 49, 50].

### The Importance of In-Person Anatomy Practical Sessions

Although our results suggest that the suspension of dissection-based teaching was not associated with a reduction in the proportion of students adopting a deep approach to learning, our results highlight the importance of maintaining exposure to cadaveric material, preferably in an in-person format. Indeed, students stated they felt disadvantaged by the lack of access to anatomy laboratories, supported by other studies which report that lack of practical sessions negatively impacted students’ confidence in anatomy [50, 51].

The benefits of in-person cadaveric teaching are well established and have previously been discussed at length [3–7]. The use of cadaveric material to teach anatomy aids in building a deep understanding of 3D relations, as well as acquiring an appreciation of natural anatomical variation [3–7]. Practical anatomy classes also provide students with additional resources for learning, such as models and clinical imaging [9, 19]. In-person teaching using cadaveric specimens also provides students with the opportunity for student-to-student and student-to-instructor interaction, thereby encouraging discussions and providing an opportunity to use anatomical terminology. Students also learn humanistic elements associated with working with human cadaveric material, such as empathy, encountering death and ethics [3, 6, 9, 10, 52–55]. These elements of the hidden curriculum in anatomical dissection classes are difficult to replicate through online means [56]. In addition, it has been reported that delivery of face-to-face anatomy practical classes led to better examination results and student satisfaction when compared to online sessions [57]. Moreover, students who adopted a deep approach to learning demonstrated strong agreement when asked whether dissecting room teaching and getting their “hands in and feeling for structures” was an effective way of learning anatomy, suggesting that active participation in cadaveric practical classes aids learning. This relationship was also reported by Smith and Mathias [5], and their conclusions were echoed by Choi-Lundberg et al. [58].

### Provision of Online Anatomy Resources

The majority of students reported that online resources (cadaveric videos, non-cadaveric videos, cadaveric images and virtual 3D models) were effective resources to learn anatomy online, mirroring results from previous studies [59]. Furthermore, for the majority of resources, the institution at which the student studied did not significantly affect opinions on their perceived effectiveness. However, there was a significant difference between opinions for online cadaveric video resources (*p* = 0.049), instructor made cadaveric videos (*p* = 0.035), virtual 3D models (*p* = 0.048) and instructor made mock examinations (*p* = 0.05) (for full details, see Table 4). This suggests that in these instances, the quality of the resource provided or the way in which the resource was integrated into curriculums may have differed, making it difficult to attribute the student’s perceived effectiveness to the resource itself. Nevertheless, results from the thematic analysis have provided a useful insight into how to improve online resources and their integration into the curriculums.

The thematic analysis revealed that students’ opinions of resources varied greatly; what worked for one student did not work for another. Academics should take this into consideration, as it mirrors previous findings that suggest using multiple pedagogical resources simultaneously is the best way to teach anatomy [6]. This is perhaps of particular importance to deep learners, who described limitations in the breadth of online resources offered by their school (Table 5).

Students clearly expressed in the open-ended responses that they desire a range of resources, balanced by the request for academics to properly signpost the relevant content and required level of detail. Thus, when combining multiple online resources, it is recommended that greater emphasis is placed on equipping students with the skills to identify the best resources for their own learning. In fact, within the learning theory of connectivism, the ability to seek and filter relevant information is a vital part of the learning process [60]. One method to achieve this is to ensure that learning objectives are explicitly stated and that students are confident using these to ensure they cover all of the core content using any of the resources of their choice. This should help to prevent students from becoming overwhelmed if a large variety of resources are provided. Another important perspective to consider here is metacognition. Flavell [61] first described metacognition as “one’s knowledge concerning one’s own cognitive processes”, encompassing the processes used by students to monitor and assess their own learning. The theory of metacognition overlaps with research surrounding self-regulated learning and self-efficacy [62–64]. It has been demonstrated that deeper approaches to learning often require student awareness of their own cognitive processes [65, 66]. Hence, if institutions provide a wide array of resources, it is essential that time is dedicated to supporting students in the development of metacognitive strategies.

### Impact of Changes to Assessment

There was very little difference in the proportions of learning approaches adopted by students regardless of whether their formative assessment(s) remained unchanged, were cancelled or were delivered online. Students attending universities that did not deliver any formative assessment prior to the initial COVID-19 lockdown were less likely to adopt a deep approach to learning online and more likely to adopt a strategic or surface approach (Fig. 1C). The benefits of providing formative quizzes in online teaching sessions have been discussed by Srinivasan [67]. Building on this, we highlight that students had a clear preference for formative examinations created by their instructors when compared to formative assessments sourced online (*p* ≤ 0.05). Embedding institution-specific formative assessments may aid with motivation [34] and can ensure students can successfully monitor their progress.

Furthermore, it is reassuring that adapting summative assessments to an online format did not appear to alter the proportion of students adopting each approach to learning (Fig. 1B). However, the move to online assessment led to increased concerns regarding the probity of examinations at an institutional level [68]. To overcome this, many universities invested in proctoring software and utilized question styles in which the answers could not be easily found online by writing questions that rewarded the application of anatomical knowledge rather than rote learning [16]. Questions written in this manner have also been shown to encourage students to adopt a deep approach to learning [5].

Where summative assessments were cancelled, the predominant learning approach adopted by students was strategic. This does not align with our hypothesis that cancelled assessments would result in smaller proportions of students adopting a strategic approach to learning. This is interesting, as strategic learners are often defined by the fact that they are intrinsically motivated by performing well in examinations. Medical students know that knowledge of anatomy is essential to becoming effective medical practitioners and hence the primary goal for these students may not be to pass the summative assessment. This could explain why cancelled summative assessments did not reduce the number of students adopting strategic approaches to learning in a medical student population [69]. Further research is needed to compare this finding with alternative student populations on less vocational degree courses.

### Study Limitations

While this is the first study of its kind to assess students’ approaches to learning anatomy online during the COVID-19 pandemic, it does have limitations, many of which arose due to the circumstances during the survey period. Data was collected at a single time-point (June–July 2020) meant it was impractical to include a control group. During the COVID-19 pandemic, online learning had a detrimental effect on students’ mental health and wellbeing, with increases in rates of depression and anxiety, and decreased satisfaction with their educational experience. These negative impacts were shown to have more significant effects on students with less disposable income, those who identify as women, non-binary or LGBTQ + , students of colour and those who also act as caregivers [70–75]. As many students were under remarkable pressures during the survey period, results presented here may not be true or applicable to other online learning environments. Finally, as a convenience sample of universities was utilized and the response rate was low, this study reflects the views of students from a limited number of universities and students in the UK and Ireland.

## Conclusion

The major conclusions from this study are highlighted below and are presented along with recommendations and practice points for educators who are responsible for the future development of blended curricula in anatomy.

1. Students continue to desire access to cadaveric material, and hence this should be maintained, where possible, during blended delivery of an anatomical syllabus. It is worth considering which specimens students need to visualise spatially in order to develop a deeper understanding and which learning outcomes of anatomy can be achieved using multimedia tools.
2. The shift to online learning may not, per se, affect the approaches to learning that students adopt. Thus, as discussions regarding blended learning approaches continue, we can provide supplemental online learning material that accompanies and complements in-person practical anatomy sessions. However, the maintenance of in-person teaching is essential, as one student exclaimed: “You can’t be a doctor by watching lectures and videos online”.
3. Formative assessments are imperative to encouraging deep learning and hence should be used to help motivate students and allow them to self-assess their own progress, thereby improving students’ metacognition.
4. Providing a range of resources is optimum when delivering a syllabus through online means. However, institutions should put equal effort into providing good practice guidelines (and/or metacognitive strategies) for those using online resources to learn from.

## Supplementary Information

Below is the link to the electronic supplementary material.

Supplementary file1 (DOCX 31 KB)

Supplementary file2 (XLSX 96 KB)

## Data Availability

All data collected is anonymous and stored on a password protected institution hosted platform.
