# Supplementary material for: A Multicentre Analysis of Approaches to Learning and Student Experiences of Learning Anatomy Online
Source: Med Sci Educ. 2022 Sep 19;32(5):1117–30. doi: 10.1007/s40670-022-01633-7 (PMC9483883; doi:10.1007/s40670-022-01633-7)
Supplement: Supplementary file 1 — Supplementary file1 (DOCX 31 KB) [file 40670_2022_1633_MOESM1_ESM.docx]

**Title:** **A Multicentre Analysis of Approaches to Learning and Student Experiences of Learning Anatomy Online**

**Authors**

Danya Stone^1^, Georga J. Longhurst^2^, Kate Dulohery^3^, Thomas Campbell^4^, Annalise Richards^5^, Dominic O’Brien^1^, Thomas Franchi^6^, Samuel Hall^8^, Scott Border^9^

Danya Stone and Georga J. Longhurst contributed equally to this work

**Affiliations**

1) Department of Medical Education, Brighton and Sussex Medical School, Brighton, East Sussex, BN1 9PX

2) Department of Anatomical Sciences, St George’s University of London, London, SW17 0RE

3) School of Medicine, University of Sunderland, Sunderland, SR1 3SD

4) School of Medicine, University College Dublin, Dublin, D04 V1W8

5) School of Anatomy, University of Bristol, Bristol, BS8 1QU

6) School of Medicine, University of Sheffield, Sheffield, S10 2TN

7) Centre for Learning Anatomical Sciences, Southampton University, SO17 1BJ

**Correspondence to: Georga J. Longhurst,** Anatomical Sciences, St George’s University of London, London, SW17 0RE, phone: 02087255230, email: [glonghur@sgul.ac.uk\](mailto:glonghur@sgul.ac.uk\)

As a follow-up to a pilot study completed in April, this survey aims to gather information on the adaptations in anatomical education due to the Covid-19 pandemic.

The survey is divided into 4 sections

1) Impact on teaching during academic year 2019/20
2) Impact on assessment for academic year 2019/20
3) Practical classes for upcoming academic year 2020/21
4) Online community for upcoming academic year 2020/21

This survey will take approximately 10 minutes to complete

Do you confirm that you have read and understand the participant information leaflet and returned the completed consent form?

- Yes
- No

Name of participant, job title and university affiliation

________________________________________________________________

On average, how many medical students are taught anatomy per academic year in your university?

________________________________________________________________

How many staff members provide anatomy teaching to medical students in your university (excluding demonstrators)?

________________________________________________________________

Which of the following applies to you

- 0 - 5 year's lecturing experience
- 5 - 10 year's lecturing experience
- 10+ year's lecturing experience

The following questions are related to emergency measures that were implemented in response to the rapid shift to online teaching due to the Covid-19 pandemic

How was anatomy lecture content delivered? Select all relevant options

- Synchronous/ live
- Asynchronous/ preprepared
- Other (please specify if not applicable) ________________________________________________

Which resources were used for synchronous/ asynchronous delivery of anatomy lecture content? Select all relevant options

- Panopto
- Echo 360
- Mediasite
- Zoom
- Big Blue Button
- Collaborate Ultra
- Microsoft Teams
- Other (please specify if not applicable) ________________________________________________

How was anatomy practical content delivered? Select all relevant options

- Digitised cadaveric resources (eg. Aclands, cadaveric images/ videos etc)
- 3D virtual resources (eg. Complete anatomy, 3D models etc)
- Other (please specify if not applicable) ________________________________________________

Which digitised cadaveric resources, if any, were used to replace practical classes? Select all relevant options

- Aclands anatomy
- Cadaveric images
- Bespoke videos of prosected/plastinated specimens
- YouTube videos
- Other (please specify if not applicable) ________________________________________________

Which anatomical 3D virtual resources, if any, were used to replace practical classes? Select all relevant options

- Visible Body
- Complete Anatomy
- Anatomy TV
- 3D models
- SECTRA
- Other (please specify if not applicable) ________________________________________________

How much do you agree or disagree with the following statements?

|  | Strongly agree | Agree | Neither agree nor disagree | Disagree | Strongly disagree | Not applicable |
| --- | --- | --- | --- | --- | --- | --- |
| I prefer delivering anatomy teaching through distance learning rather than face-to-face |  |  |  |  |  |  |
| I upskilled in new technologies to adapt to distance learning |  |  |  |  |  |  |
| My academic workload increased to adapt to distance learning |  |  |  |  |  |  |

Will resources created for distance learning during the lockdown be used to supplement teaching once face-to-face learning is resumed?

- Yes
- No
- Some resources will be used
- Other (please specify if not applicable) ________________________________________________

The following questions are related to anatomy assessment during the Covid-19 lockdown.

Please select the most appropriate answer regarding the following questions about anatomy assessment

|  | Yes | No | Not applicable |
| --- | --- | --- | --- |
| Were formative assessments delivered online? |  |  |  |
| Were summative assessments delivered online? |  |  |  |

How did you format assessments? Select all relevant options. If assessments were spotter style, please select multiple choice/ single best answer or short answer as appropriate

|  | Multiple choice/ Single best answer | Short answer | Long answer/ essays | Viva/ Oral | OSCE | None - assessments were cancelled | Not applicable |
| --- | --- | --- | --- | --- | --- | --- | --- |
| Written assessment (formative) |  |  |  |  |  |  |  |
| Written assessment (summative) |  |  |  |  |  |  |  |
| Practical assessment (formative) |  |  |  |  |  |  |  |
| Practical assessment (summative) |  |  |  |  |  |  |  |

Did summative assessment format differ from first semester?

|  | Yes | No | Not applicable |
| --- | --- | --- | --- |
| Written assessment |  |  |  |
| Practical assessment |  |  |  |

Were summative assessments closed book exams?

|  | Yes | No | Not applicable |
| --- | --- | --- | --- |
| Written assessment |  |  |  |
| Practical assessment |  |  |  |

Were students monitored during summative assessments?

|  | Yes | No | Not applicable |
| --- | --- | --- | --- |
| Written assessment |  |  |  |
| Practical assessment |  |  |  |

How much do you agree or disagree with each of the following statements?

|  | Strongly agree | Agree | Neither agree nor disagree | Disagree | Strongly Disagree | N/A |
| --- | --- | --- | --- | --- | --- | --- |
| I prefer the delivery of assessment online rather than standard assessment procedures |  |  |  |  |  |  |
| I found it easy to protect assessments against collaborative answering/cheating |  |  |  |  |  |  |

Please identify any specific challenges you experienced in delivering anatomy assessments online

________________________________________________________________

Please identify any specific opportunities you identified in delivering anatomy assessments online

________________________________________________________________

The following questions are related to procedures put in place for the next academic semester

In terms of the next academic semester, do you agree or disagree with each of the following statements?

|  | Yes | No | Undecided | N/A |
| --- | --- | --- | --- | --- |
| I will continue to deliver **all** anatomy lecture content online |  |  |  |  |
| I will continue to deliver **all** anatomy practical content completely online |  |  |  |  |
| I will continue to host **all** anatomy assessments online |  |  |  |  |
| I will implement additional measures to protect against collaborative answering/ cheating in online assessments |  |  |  |  |

Please tick the most appropriate statement in relation to your donor body program

- Suspended until further notice
- Suspended with a confirmed date to resume (specify date) ________________________________________________
- Resumed (specify date)
- Unaffected

Are you facing a reduction in the number of cadaveric specimens available for teaching?

- Yes
- No
- Other (please specify if not applicable) ________________________________________________

If you answered 'yes' to the above question, do you think this will have an impact on anatomy teaching in the future? Are there any plans on how to compensate for this?

________________________________________________________________

Will medical students perform anatomy dissection in the next academic semester?

- Yes
- No
- Undecided
- No - students did not perform dissection pre Covid-19 lockdown

Will medical students have physical access to cadaveric material in the next academic semester?

- Yes
- No
- Undecided
- No - students did not have access to cadaveric material pre Covid-19 lockdown

Which adaptations will be implemented to facilitate anatomy practical classes. Please select all relevant options

- Extra personal protective equipment (PPE) will be provided
- Class sizes will be reduced
- Social distancing will be enforced (1m or 2m depending on current guidelines)
- Student numbers per donor or cadaveric specimen will be restricted
- Practical classes are cancelled
- Other (please specify if not applicable) ________________________________________________

Regarding the next academic semester, how much do you agree or disagree with the following statements?

|  | Strongly agree | Agree | Neither agree nor disagree | Disagree | Strongly disagree |
| --- | --- | --- | --- | --- | --- |
| I think that adapting dissection activities/practical classes will be easily executed |  |  |  |  |  |
| I think that adaptations to dissection activities/practical classes will improve student learning |  |  |  |  |  |
| I think that adaptations to dissection activities/practical classes will improve student-student interactions |  |  |  |  |  |
| I think that adaptations to dissection activities/practical classes will improve student-tutor interactions |  |  |  |  |  |

Please identify any specific challenges your university will face in implementing adaptations to dissection activities/practical classes

________________________________________________________________

Please identify any specific opportunities in implementing adaptations to dissection activities/practical classes

________________________________________________________________

Has your university purchased virtual anatomy licences for the next academic semester?

- Yes (specify) ________________________________________________
- No
- Planning on doing so
- Would like to but restricted by budget

How much do you agree or disagree with the following statements?

|  | Strongly agree | Somewhat agree | Neither agree nor disagree | Somewhat disagree | Strongly disagree | Not applicable |
| --- | --- | --- | --- | --- | --- | --- |
| I prefer working from home than on site |  |  |  |  |  |  |
| I think staff interactions are better from working from home rather than on site |  |  |  |  |  |  |
| I think student engagement will improve with continued online teaching in the next academic semester |  |  |  |  |  |  |

Has the Covid-19 lockdown led to new academic collaborations for you?

- Yes (specify) ________________________________________________
- No

Does your university have a strategy in place to increase peer-to-peer communications for the next academic semester?

- Yes (specify) ________________________________________________
- No
- Other (please specify if not applicable) ________________________________________________

Does your university have a strategy in place to increase peer-to-tutor communications for the next academic semester?

- Yes (specify) ________________________________________________
- No
- Other (please specify if not applicable) ________________________________________________
